# Anxiety and Depression Among Patients With Irritable Bowel Syndrome in Hail, Saudi Arabia: Towards a Better Understanding of the Psychological Role

**DOI:** 10.7759/cureus.73290

**Published:** 2024-11-08

**Authors:** Mohammed Salem Alharbi, Renad Meshal Alharbi, Maha Qasem Almutairi, Farida Habib Khan, Ethar Abed Alsulami, Abdulaziz Mohammed Alenezi

**Affiliations:** 1 Department of Medicine, University of Hail College of Medicine, Hail, SAU; 2 Department of Medicine, King Faisal Specialist Hospital and Research Centre, Riyadh, SAU; 3 Department of Medicine, King Saud University Medical City, Riyadh, SAU; 4 Department of Community Medicine, University of Hail College of Medicine, Hail, SAU; 5 Department of Obstetrics and Gynecology, East Jeddah Hospital, Jeddah First Health Cluster, Jeddah, SAU; 6 Department of Neurology, King Fahad Medical City, Riyadh, SAU

**Keywords:** anxiety, depression, irritable bowel syndrome, psychology, saudi arabia

## Abstract

Introduction: Patients with irritable bowel syndrome (IBS) often experience psychiatric disorders such as anxiety and depression. This study examined the association between IBS and anxiety and depression and explored how these variables interact with various IBS indices in the general population of Hail, Saudi Arabia.

Methods: This cross-sectional descriptive study utilized standardized assessments for diagnosing IBS, anxiety, and depression. An online questionnaire was distributed to individuals in the Hail region.

Results: A total of 687 participants completed the questionnaire, of whom 508 (74%) were female. According to IBS diagnostic criteria, only 176 (26%) of participants had IBS. Based on the patient health questionnaire (PHQ) criteria for depression, 497 (72%) of participants were identified as depressed. Similarly, the generalized anxiety disorder (GAD) criteria indicated that 633 (92%) of participants suffered from GAD. The diagnosis of IBS was significantly associated with GAD, depression, and psychological symptoms such as annoyance, irritability, and concern about daily tasks.

Conclusion: There is a significant association between IBS and anxiety and depression, suggesting that psychiatric disorders should be routinely screened for in patients with IBS. Recognizing and treating these comorbidities can improve outcomes for individuals with IBS.

## Introduction

Irritable bowel syndrome (IBS) is a functional disorder characterized by chronic abdominal pain and altered bowel habits, often related to defecation. According to the predominant complaint, it can be categorized as constipation-predominant IBS (IBS-C), diarrhea-predominant IBS (IBS-D), and mixed IBS (IBS-M). Patients who do not fit into the first three subgroups despite meeting diagnostic criteria are identified as having unclassified IBS (IBS-U) [1]. The global prevalence of IBS is approximately 11.2%. The prevalence of IBS varies from one country to another and also according to the criteria used for diagnosis [2]. In Northern Saudi Arabia, the prevalence is reported as 11% among males and 12.5% among females, based on the latest Rome IV criteria [3]. The ROME IV criteria define IBS based on recurrent abdominal pain associated with altered bowel habits. According to these criteria, a diagnosis of IBS requires abdominal pain occurring at least one day per week over the past three months, with the onset of symptoms at least six months prior. This pain must be associated with two or more of the following: changes in the frequency of bowel movements, changes in stool form or appearance, and relief or worsening with defecation [1]. Notably, the pathophysiology of IBS remains unclear. Suggested mechanisms include genetic factors [4], abnormal motor function of the gastrointestinal tract [5,6], psychopathology [7,8], visceral hypersensitivity [9], and the consequences of infectious gastroenteritis [10]. Psychological stress not only contributes directly to IBS symptoms but may also indirectly affect intestinal motility [11].

The literature suggests a link between IBS and neuropsychiatric disorders such as anxiety and depression, although the exact relationship remains unclear and varies in its manifestations [12,13]. Remarkably, two studies reported that anxiety may reduce the risk of developing IBS, though these findings were not statistically significant [14,15]. A systematic review and meta-analysis by Sibelli et al. found that the risk of IBS onset doubled in patients who self-reported anxiety and depression. However, definitive diagnoses of these psychological comorbidities provide limited evidence, as most studies indicated that scores measuring generalized distress, irrespective of clinically confirmed diagnoses, were a good predictor of IBS onset [13]. A meta-analysis by Fond et al. indicated no significant association between psychological comorbidities and specific IBS subtypes, nor between IBS-C and depression. This was attributed to the limited number of studies included. Hence, further research with higher power is warranted [12].

A previous study successfully demonstrated a possible link between 5-hydroxytryptamine or serotonin (5-HT) levels and different IBS subtypes. Patients with IBS-C showed impaired postprandial 5-HT release, while levels were significantly higher in controls and IBS-D patients [16]. Patients with IBS experiencing anxiety or depression often report more severe gastrointestinal symptoms and a lower quality of life [17-19]. In Saudi Arabia, research has shown that morbid anxiety is a predictor of IBS among nurses [20]. Anxiety and depression have been demonstrated to be significantly associated with IBS in Northern Saudi Arabia. However, this association was only investigated using patients’ self-reports of anxiety, depression, or both [3]. Therefore, further investigation using standardized assessments is essential in this region. A multicenter study in Riyadh found that 44% of female patients and 32% of male patients attending gastroenterology clinics exhibited depressive symptoms, while 34% of females and 24% of males experienced anxiety symptoms [21].

Psychological factors are thought to contribute to the pathogenesis of IBS [8,11]. Nevertheless, they often appear complicated, with vague, unpredictable, and specific manifestations. Therefore, more descriptive studies are warranted. This study aims to investigate the association between IBS and anxiety and depression among patients in the general population of Hail, Saudi Arabia, and to describe their interactions with various IBS indices.

## Materials and methods

This cross-sectional descriptive study was conducted from 2020 to 2021 and planned for the mid-2020 period. The Research Ethics Committee (REC) of the University of Hail obtained ethical approval for the proposal in October 2020.

The study population included adolescents (>15 years) and adults residing in both urban and rural areas of Hail, Saudi Arabia. According to recent literature, up to 13% of the population in Northern Saudi Arabia has been found to have IBS [3]. Participants were sampled with a confidence interval (CI) of 95%.

The following formula was used: N = Zα/22 * p̂*(1- p̂)/ε^2^

At a confidence interval of 95%, Zα/2 is equal to 1.96. The margin of error(ε) was set at 0.05, and the estimated population proportion (p̂) is 0.13. This formula yielded a population size (N) of 173.7.

N = Zα/22 * p̂*(1- p̂)/ε^2^

N = (1.96)2 x 0.13 x (1- 0.13)/(0.05)^2^

N = 3.84 x 0.13 x 0.87/0.0025

N= 173.7

Therefore, researchers planned to enroll more than 174 participants in the study to increase its power (power = 1 - β). Ultimately, 687 participants were included in the study.

Data were collected by using a self-administered online questionnaire that employed standardized assessments for diagnosing IBS, anxiety, and depression. The ROME IV Criteria were utilized for diagnosing IBS [22]. A validated Arabic version of the PHQ was used to separately assess depression and generalized anxiety disorder [22]. After providing consent to participate in this study, participants were asked to fill out a questionnaire comprising four domains. The first domain collected demographic data, including age, sex, marital status, socioeconomic status, educational level, working hours, residence, and nationality. The second domain gathered relevant general health information, such as smoking habits, height, weight, exercise frequency, and caffeine consumption. The third domain focused on psychological indices, specifically inquiring about any previous diagnoses of psychological disorders other than anxiety or depression and whether participants were on medications for underlying psychiatric conditions, along with the PHQ for depression and generalized anxiety disorder (GAD). The fourth domain contained questions aimed at diagnosing IBS using the ROME IV criteria, classifying IBS into specific subtypes, and measuring symptom severity. In the third and fourth domains, participants were asked to self-report in addition to their diagnoses via standardized assessments. The questionnaire was initially sent to random participants for pilot testing to confirm that no ambiguous questions existed and to ensure that it was fully clear, understandable, and suitable for participants.

The questionnaire was supplemented with a cover letter detailing the research objectives, inclusion criteria, confirmation of confidentiality, informed consent, and the principal investigator’s contact information. Informed consent was obtained from all participants before their enrollment in the study.

Data were entered and coded using Microsoft Excel, and Statistical Package for Social Sciences (SPSS) version 23 was used for descriptive and inferential analyses (IBM Corp., Version 23.0. Armonk, NY). Descriptive analyses are presented as frequencies and percentages, while the chi-square test was employed for statistical analysis. The level of significance (p-value) was set at ≤ 0.05.

## Results

In this study, the majority of respondents, 508 (74%), were female. Table 1 outlines the demographic characteristics of the participants. A significant portion, 414 (60%), were young adults aged between 15 and 20 years, while 569 (83%) resided in Hail City. In terms of education, 317 (47%) held a bachelor’s degree. Furthermore, 173 (25%) of the participants were classified as overweight, and 151 (21.5%) fell into the obese category.

**Table 1:** Demographic profile of the respondents (n=687)

Variable	N	%
Age in years		
15-25	414	60
26-35	106	15
36-45	98	14
46-55	57	9
56 and above	6	1
Missing	6	1
Residents		
Hail	569	83
Provisional and Neighboring Villages	44	6
Outside Hail	68	10
Missing	6	1
Current educational level		
Elementary	13	2
High School	300	43
Bachelor	317	47
Higer education	29	4
Others	20	3
Missing	8	1
BMI (kg/m^2^)		
(16-17-Moderate Thinness)	13	2
(17-18.5- Mild Thinness)	48	7
(18.5-25-Normal)	279	41
(25-30-Overweight)	173	25
(30-35-Obese Class-1)	98	14
(35-40-Obese Class-2)	43	6
(40-70-Obese Class-3)	10	1.5
Missing	23	3.5

As shown in Table 2, 372 (54%) participants did not engage in any exercise. Among those who exercised, 85 (13%) exercised for about 30 minutes. Furthermore, 623 (90.5%) participants were non-smokers, while a small minority, 38 (5.5%), identified as smokers. Of those smokers, 9 (14%) reported smoking 16-20 cigarettes per day. In terms of caffeine consumption, 360 (52%) participants consumed 2-4 cups of caffeinated drinks daily, whereas 156 (23.5%) consumed 5-12 cups per day.

**Table 2:** Habit profile of the respondents

Variable	N	%
Exercise days per week		
1-2 days	146	21
3-4 days	85	13
5-6 days	51	7
Every day	27	4
Do not exercise	372	54
Missing	6	1
Duration of exercise per day		
10-20 minutes	80	11.5
Around half an hour	85	13
Around 45 minutes	67	10
Around an hour	51	7
Around 1 and a half hour	22	3
Around 2 hours	4	0.5
Do not exercise	372	54
Missing	6	1
Smoking status		
Smoker	38	5.5
Ex-Smoker	27	3
Non-Smoker	623	90.5
Missing	6	1
Cigarettes consumed/day		
1-5	31	48
6-10	9	14
11-15	8	12
16-20	9	14
21-25	2	4
26 or more	6	9
Years of smoking		
1-4	40	63
5-10	6	9
11-14	8	12
15-20	7	11
21-24	1	0.5
25-30	3	4.5
Daily average consumption of caffeinated drinks		
One cup/day	161	23
Two-four cups/day	360	52
Five-seven cups/day	126	19
Ten-twelve cups/day	30	4.5
Missing	10	1.5

Abdominal pain that woke participants from sleep as the only red flag symptom was reported by 123 (18%) respondents, while 100 (14.5%) experienced unintentional weight loss, as shown in Table 3. Moreover, 69 (10%) of participants reported experiencing both symptoms simultaneously. Table 3 also highlights other combinations of red flag symptoms.

**Table 3:** Red flags experienced by the respondents Respondents had multiple options to select.

Symptom	N	%
Unintentional weight loss	100	14.5
Unintentional weight loss, and abdominal pain awakening from sleep	69	10
Unintentional weight loss, abdominal pain awakening from sleep , and bloody diarrhea	30	4
Unintentional weight loss, and bloody diarrhea	31	4.5
Abdominal pain awakening from sleep	123	18
Abdominal pain awakening from sleep, and bloody diarrhea	27	4
Bloody diarrhea	55	8
Does not complain of any symptom	352	51

Table 4 illustrates that when participants were asked if they believed they had any psychological ailment, only 126 (18%) responded affirmatively. Among these, only 17 (13%) of participants were taking psychiatric medication. GAD and depression were the most common complaints, affecting 40 (32%) and 32 (25%) of respondents, respectively.

**Table 4:** Psychological illness of the respondents

Variable	N	%
Do you have any psychological illness? (n=687)		
Yes	126	18
No	555	81
Missing	6	1
What type of psychological illness do you have? (n=126)		
Depression	32	25
Generalized anxiety disorder	40	32
Panic attacks	12	9.5
Obsessive compulsive disorder	19	15
All of above	17	13.5
Missing	6	5
Do you use any type of psychiatric medication? (n=126)		
Yes	17	13
No	103	82
Missing	6	5

When respondents were queried about their psychological status over the last four weeks (Table 5), 170 (25%) of them reported becoming easily bothered and irritable over minor issues, while 150 (22%) worried excessively about daily matters. A total of 123 out of 681 participants felt nervous and anxious at all times, with symptoms occurring daily.

**Table 5:** Psychological status of the respondents during the last four weeks (missing=6)

Variable	Not at all	Several days	More than half the days	Nearly every day
Feeling nervous, or anxious	145	282	131	123
Not being able to stop/control worrying	237	221	107	116
Worrying too much about different things	176	228	127	150
Trouble relaxing	246	227	112	96
Being so restless that it is hard to sit still	398	161	62	60
Getting easily annoyed or irritable	177	217	117	170
Feeling frightened as if something terrible might occur	323	166	89	103
Difficult to do work, take care of things at home, or get along with people	266	298	78	39

New symptoms experienced by respondents during the last two weeks while completing the study questionnaire included difficulty falling asleep, staying asleep, or sleeping too much, reported by 139 (25%) of the participants. Additionally, 117 (17%) of respondents reported feeling tired and experiencing reduced appetite or overeating, as shown in Table 6.

**Table 6:** New symptoms, which the respondents have experienced during the last two weeks (missing=6)

Symptom	Not at all	1-2 days	More than 3 days	Almost every day
Little interest in doing things	237	264	98	82
Feeling depressed or hopeless	237	264	98	82
Disturbance in falling or staying asleep, or sleeping excessively	190	231	121	139
Feeling tired	180	224	160	117
Poor appetite or overeating	180	224	160	117
Feeling bad about yourself or that you are a failure	340	147	94	100
Trouble in concentrating on things	325	199	75	82
Moving or speaking so slowly that other people have noticed	471	127	36	47
Thoughts that you would be better off dead or thoughts of hurting yourself	520	87	35	39

Regarding gastrointestinal tract (GIT) complaints (Table 7), 379 (55%) of participants reported experiencing weekly abdominal pain, with 500 (72.5%) stating that their pain improved after defecation. The majority, 434 (63%), of participants indicated that bowel movements were intensified with the onset of abdominal pain. Additionally, 344 (50%) experienced mixed types of stool, characterized as occasionally loose, while others were hard. 

**Table 7:** Gastrointestinal tract (GIT) complaints and Rome IV criteria for IBS

Variable	N	%
Frequency of abdominal pain		
Once or more every week	379	55
Not often	302	44
Missing	6	1
Relationship of abdominal pain with defecation		
Worsen	179	26
Improves	500	72.5
Missing	8	1.5
Change in bowel movements with the onset of abdominal pain		
Remains as usual	243	35
Intensifies	434	63
Missing	10	2
What is the usual pattern of your stool?		
Diarrhea	90	13
Constipation	96	14
Mixed	344	50
Missing	157	23

**Figure 1:**
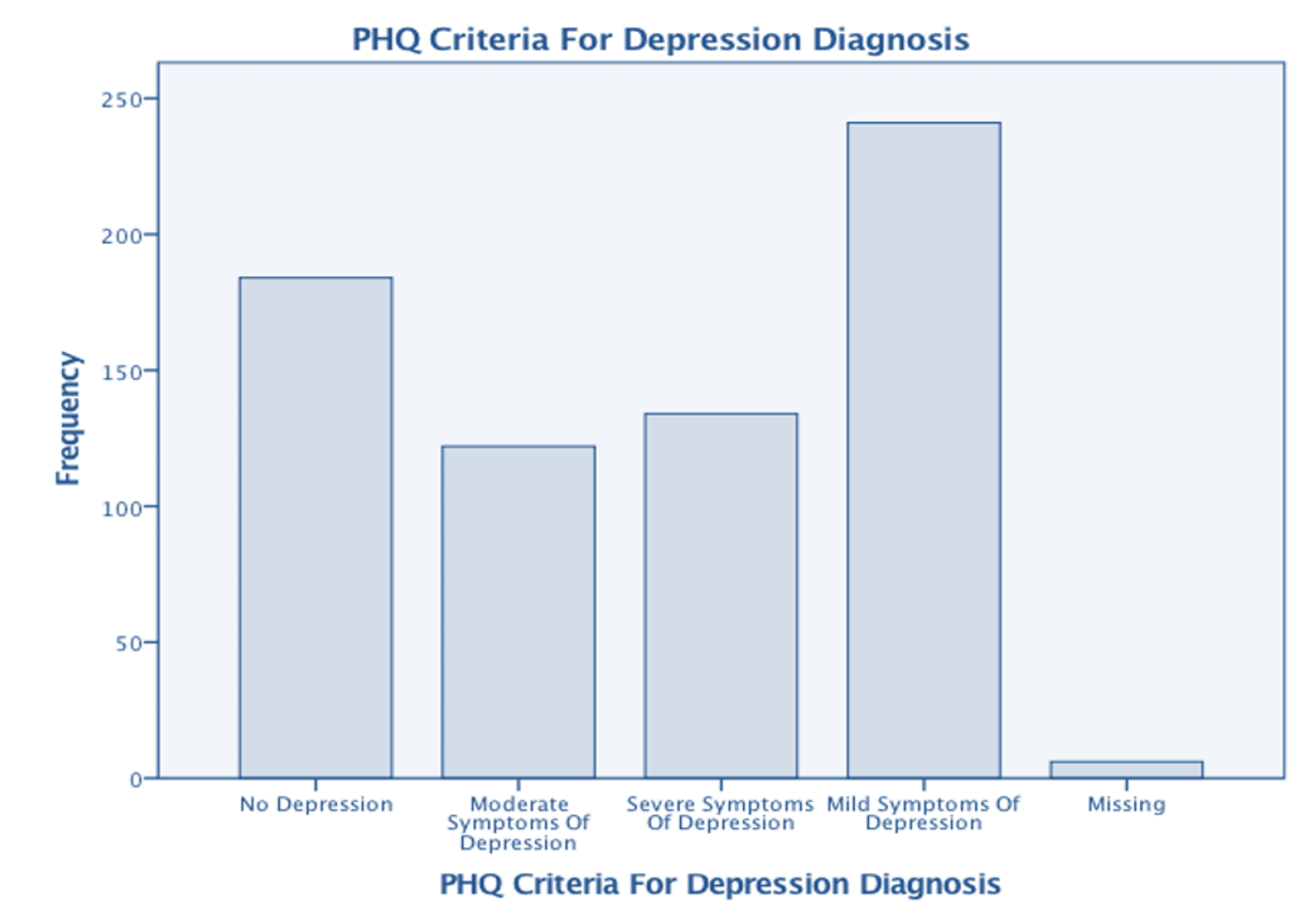
PHQ criteria for the depression diagnosis (n=687) PHQ: patient health questionnaire 0-4= No depression, 184 (27%) 5-9= Mild symptoms of depression, 241 (35%) 10-14= Moderate symptoms of depression, 122 (18%) 15 or more= Severe symptoms of depression, 134 (19%) Missing= 6 (1%)

**Figure 2:**
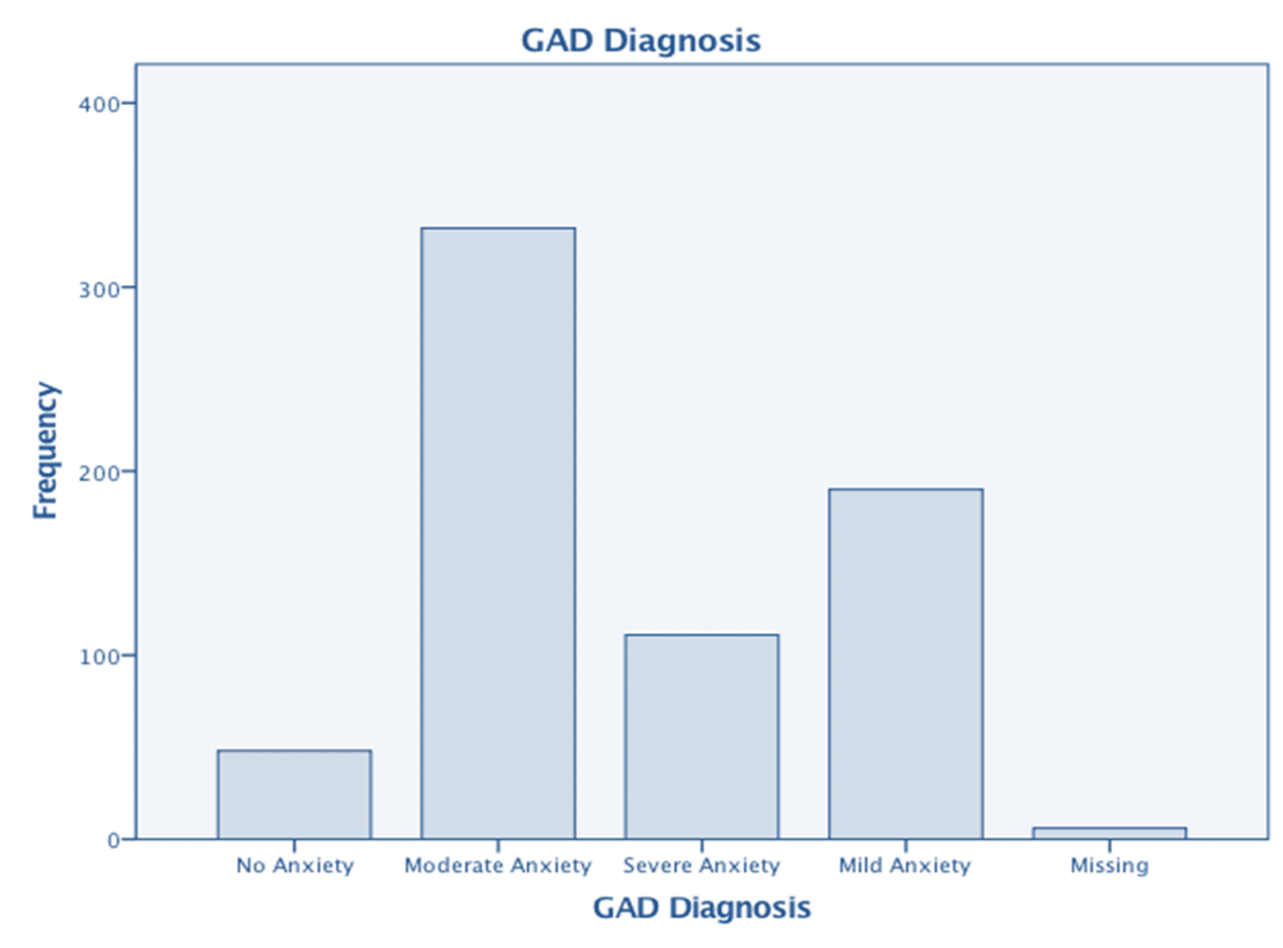
GAD diagnosis (n= 687) GAD: generalized anxiety disorder 0-4= No anxiety, 48 (7%) 5-9= Mild anxiety, 190 (28%) 10-14= Moderate anxiety, 332 (48%) 15 or more= Severe anxiety, 111 (16%) Missing= 6 (1%)

Table 8 presents the findings of this study. It reveals that only 190 (28%) of participants admitted to feeling depressed; however, when the PHQ criteria for depression were applied (Figure 1), all participants, except for 184 (27%), were classified as depressed, with 134 (19%) categorized as severely depressed. Similarly, according to the criteria for GAD diagnosis, all participants, except for 48 (7%), had mild to severe anxiety, as shown in Figure 2. Among the responses received, 6 (1%) of responses were recorded as missing values. According to participants’ perceptions, 348 (51%) believed they had IBS, and 333 (48%) reported having some form of psychological issue. Respondents indicated that psychological problems appeared first in 167 (24%) of cases, while symptoms of IBS were reported first in 145 (21%) of cases.

**Table 8:** Probable diagnosis by the respondents (self report)

Variable	N	%
State of depression by the respondents		
I am depressed	190	28
I am not depressed	491	71
Missing	6	1
Probable diagnosis by the respondents themselves		
I have IBS	348	51
I have a psychological problem	333	48
Missing	6	1
Symptoms that appeared first, according to the respondents		
Irritable bowel syndrome	145	21
Psychological problems	167	24
Not certain	272	40
Missing	103	15

**Figure 3:**
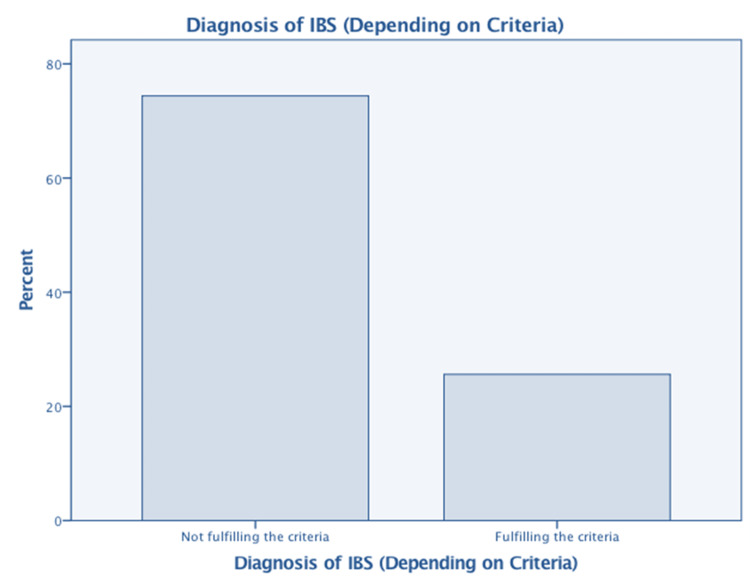
Diagnosis of IBS (Depending on Criteria) IBS: irritable bowel syndrome False (not fulfilling the criteria)=505 (73%) True (fulfilling the criteria)=176 (26%) Missing= 6 (1%)

Figure 3 presents the diagnosis of IBS based on the specified criteria. Among the 681 participants, 176 (26%) were diagnosed with IBS. Table 9 shows the association between IBS diagnosis and several relevant variables. A highly significant association (p-value=0.000) was found between IBS and conditions such as GAD, depression, and psychological symptoms, including irritability, excessive worry about daily tasks, and difficulty relaxing. Additionally, IBS was significantly associated with a body mass index (BMI) greater than 30 and female gender (p-value= 0.001 and 0.012, respectively). However, the association with caffeine consumption defined as more than five cups per day was not significant (p-value=0.562), potentially due to confounding factors such as the caffeine concentration in beverages, as the Saudi population typically consumes drinks with lower caffeine content.

**Table 9:** Association of diagnosis of IBS with relevant variables The chi-square test was applied keeping the level of significance ≤ 0.05

Comparing variable	Diagnosis of IBS (those fulfilling criteria)
	p-value
GAD diagnosis	0.000
Female gender	0.012
Taking caffeinated drinks more than 5 cups/day	0.562
With a BMI of more than 30	0.001
Annoyed or irritable	0.000
Worried too much about daily tasks	0.000
Trouble relaxing	0.000
Depression diagnosis	0.000

## Discussion

The psychological disorders experienced by patients with IBS warrant thorough assessment across all societies. In Northern Saudi Arabia, a significant association exists between IBS and anxiety and depression. However, this relationship has primarily been investigated through patients' self-reports of anxiety, depression, or both [3]. The necessity of psychiatric evaluation in patients with IBS has been emphasized in the literature on functional gastrointestinal disorders [23]. This study aimed to examine the relationship between anxiety and depression in patients with IBS and how these conditions interact with various IBS indices in the general population of Hail, Saudi Arabia.

In our study, the majority meeting the Rome IV criteria were female, which may be attributed to a higher prevalence of psychological disorders among females [24]. Alosaimi et al. revealed that females reported more symptoms of depression and anxiety than males [21]. Furthermore, 334 (49%) of respondents indicated experiencing red flag symptoms, highlighting the importance of excluding any organic causes when diagnosing IBS. Our findings suggest that emotional stress, psychiatric disorders (including anxiety and depression), and dietary habits are more common among those aged 15-25 [3]. We observed that 348 (51%) of participants believed they had IBS, whereas only 176 (26%) met the Rome IV criteria. This aligns with a study conducted in Hail [3], which reported a prevalence of approximately 23.5%, while a study in Jeddah [20] found 14.4%. The slight variations in prevalence may be attributed to cultural or dietary differences, as well as sample size, age groups, and diagnostic criteria used in various studies [25].

Regarding anxiety disorders, only 40 (5.8%) of participants believed they had anxiety, while 633 (92%) met the GAD diagnosis criteria. Additionally, most patients with IBS in this study met the GAD diagnosis criteria. In comparison, Irina’s study [17] found that 44.9% of patients with IBS had anxiety disorders. Furthermore, a study conducted in Riyadh indicated that 58% of patients with IBS experienced anxiety symptoms [21]. Out of 687 participants, 491 (71%) assumed they did not have a depressive disorder, while 497 (72%) actually met the criteria. There was a significant association between those who met the Rome IV criteria and the presence of depression. In addition, a study conducted among gastroenterology patients indicated that 76% exhibited depressive symptoms, while Irina’s study [17] found that 25.7% of patients with IBS had depression. Remarkably, when we inquired whether psychological symptoms or IBS symptoms appeared first, many respondents expressed confusion, reflecting the complex interplay between IBS and psychological disorders. Furthermore, this discrepancy between self-perception and the reality of anxiety or depression may stem from stigma and the tendency to deny psychological symptoms [26].

The increased prevalence of depression and anxiety among patients fulfilling IBS criteria may explain why some individuals improve when prescribed antidepressants and anxiolytics [27,28]. This underscores the need for further research into which antidepressants and anxiolytics are most effective for patients with IBS. The association between IBS and psychological disorders, including anxiety and depression, is well established. Our findings corroborate this relationship, indicating a strong association between IBS and anxiety/depression. Multiple studies worldwide have demonstrated that individuals with IBS and high levels of anxiety/depression experience an increased incidence of IBS symptoms compared to their peers [3,13,17,18,25].

A key limitation of this study is its observational design, which limits the ability to draw definitive conclusions about the relationship between IBS and psychological factors like anxiety and depression. Future studies, including randomized controlled trials (RCTs), are needed to more thoroughly assess the effectiveness of interventions targeting both IBS and its associated psychological symptoms. Moreover, there was a variation in the gender ratio among the respondents. We suggest conducting additional studies with a balanced sample of both males and females to further explore potential differences.

## Conclusions

In conclusion, our study highlights a significant association between IBS and mental conditions such as anxiety/depression among patients in Hail, Saudi Arabia. The findings reveal that more than half of the participants had moderate to severe anxiety symptoms, and a significant proportion also met the criteria for depression, with nearly one-fifth classified as severely depressed. This strong correlation between IBS and psychological conditions emphasizes the critical need to address mental health issues as an integral component of IBS management. The psychological distress experienced by these patients may not only exacerbate their IBS symptoms but also contribute to the overall burden of the condition.

Additionally, the data indicate that psychological problems sometimes precede the onset of IBS symptoms, suggesting a potential role for anxiety and depression in the development or worsening of IBS. This emphasizes the importance of incorporating mental health interventions early in the treatment process for IBS patients. By addressing anxiety and depression, healthcare providers can potentially improve both the psychological and gastrointestinal outcomes for these patients, leading to more comprehensive and effective management of IBS. The significant association between IBS and psychological factors highlights the need for a multidisciplinary approach to treatment, combining both gastrointestinal and mental health care.
